# Understanding breast cancer as a global health concern

**DOI:** 10.1259/bjr.20211033

**Published:** 2021-12-14

**Authors:** Louise Wilkinson, Toral Gathani

**Affiliations:** 1 Oxford Breast Imaging Centre, Oxford University Hospitals NHS Foundation Trust, Oxford, UK; 2 Nuffield Department of Population Health, University of Oxford, Oxford, UK; 3 Department of Surgery, Oxford University Hospitals NHS Foundation Trust, Oxford, UK

## Abstract

Breast cancer is now the most commonly diagnosed cancer in the world. The most recent global cancer burden figures estimate that there were 2.26 million incident breast cancer cases in 2020 and the disease is the leading cause of cancer mortality in women worldwide. The incidence is strongly correlated with human development, with a large rise in cases anticipated in regions of the world that are currently undergoing economic transformation. Survival, however, is far less favourable in less developed regions. There are a multitude of factors behind disparities in the global survival rates, including delays in diagnosis and lack of access to effective treatment. The World Health Organization’s new Global Breast Cancer Initiative was launched this year to address this urgent global health challenge. It aims to improve survival across the world through three pillars: health promotion, timely diagnosis, and comprehensive treatment and supportive care. In this article, we discuss the key challenges of breast cancer care and control in a global context.

## Introduction

Breast cancer represents a significant global health challenge: it is the most commonly diagnosed cancer in the world with an estimated 2.26 million cases recorded in 2020 and is the leading cause of cancer mortality among females. Although historically considered to be a disease of largely developed countries, over half of breast cancer diagnoses and two-thirds of breast cancer related deaths occurred in the less developed regions of the world in 2020.^1^ This article explores the reasons for the observed variations in the global burden of breast cancer and the challenges of effective care and control.

## Breast cancer incidence

Breast cancer incidence is highly correlated with human development. The human development index is a composite measure of life expectancy, education and wealth and is a more useful comparator between countries than income alone.^2^ Countries with the highest levels of human development have the highest incidences of breast cancer.^1^ The global age standardised incidence rate in females is estimated to be 48/100,000, varying from under 30/100,000 in sub-Saharan Africa to over 70/100,000 in Western Europe and North America. Although the relative incidence of breast cancer is highest in the most developed regions of the world, much larger populations in less developed regions mean that over half of all breast cancer cases are diagnosed in low- and middle-income countries, creating a significant burden of disease.

The observed global variations in the incidence of breast cancer need to be considered in the context of the known risk factors for the disease. Age is the most important risk factor and the highest age-specific incidence rates are observed in the oldest females.^3^ In the UK, over a third of breast cancer occurs in females over the age of 70, and less than one in five females are under the age of 50 at diagnosis. In less developed countries, by contrast, over half of breast cancer occurs in females under the age of 50. A younger population and a decade shorter life expectancy is the main driver of the average younger age at presentation in less developed countries.^4^ As life expectancy increases alongside economic development in these regions, we can expect to see an increase in breast cancer incidence.^1^

Other risk factors of importance can be classified into reproductive and non-reproductive factors, which are all influenced by economic development. Breast cancer risk increases with younger age at menarche, older age at menopause, having fewer children and less exposure to breast feeding.^5,6^ Increased levels of human development tend to lower the average age at menarche by improving average nutritional status which is a key determinant of age of onset of menarche. Non-reproductive risk factors of particular interest for breast cancer include obesity,^7^ with a doubling of breast cancer risk observed in overweight post-menopausal females, and increased alcohol consumption which is estimated to contribute to approximately 4% of all breast cancer cases diagnosed in 2020.^8^ Approximately 5–10% of breast cancers have an underlying genetic or hereditary cause such as BRCA1 or BRCA2 mutations, but eight out of nine females who are diagnosed with breast cancer do not have an affected female first degree relative.^9^

Studies examining breast cancer incidence in migrant populations in indigenously white countries suggest that the observed lower breast cancer incidence in ethnic minority females is largely explained by variations in the prevalence of known risk factors for the disease rather than any inherent protection against the disease in these groups.^10^ As countries in less-developed regions of the world undergo socio-economic transition, factors such as increasing life expectancy, changes in female reproductive patterns, rising obesity rates and other lifestyle associated risk factors will contribute significantly to increasing breast cancer incidence rates.^11^

## Breast cancer survival

In 2020, breast cancer was responsible for almost 685,000 deaths in females worldwide. Almost two-thirds of those deaths were recorded in less-developed regions. In more developed regions, overall 5-year survival from breast cancer is well over 80%; in comparison, 5-year survival in India is reported as less than 70% and less than 50% in South Africa.^1^ The observed survival advantage for patients diagnosed with breast cancer in more developed countries can be largely attributed to a combination of early detection strategies, access to early diagnosis and better access to effective treatments.^12^ By contrast, delayed presentation is more common in less developed regions of the world, with over half of breast cancers being locally advanced or metastatic at presentation.

The reasons for delays in presentation are multifactorial and likely arise due to a combination of low levels of cancer literacy in communities and health workers, coupled with complex and fragmented health care systems which are difficult to navigate in large part due to financial constraints.^13^ More advanced disease at presentation not only leads to poorer survival outcomes for individuals but often requires more extensive and expensive treatment which may not be readily available, placing further stress on already fragile healthcare systems.^14^

In response to the latest global cancer statistics, the World Health Organisation launched the new Global Breast Cancer Initiative in 2021 to comprehensively address the global health challenge of breast cancer, aiming to improve survival rates globally through the three pillars of health promotion, timely presentation and diagnosis, and comprehensive treatment and supportive care.^15^ Health promotion focuses on community-based education around symptom presentation and healthcare provision, addressing cancer stigma and developing risk modification strategies such as tackling obesity. Community health workers are an essential component of healthcare systems in poorer countries. They are usually lay individuals whose core functions often include health education and local ownership of national health programmes, as well raising community awareness about breast cancer to promote earlier diagnosis which is key to timely presentation and diagnosis.

The increasing complexity of diagnosis and treatment of breast cancer presents challenges in all resource settings. Current best practice has been established within highly developed healthcare systems. As the burden of breast cancer increases in low- and middle-income countries, there is an increasing need to understand the challenges of diagnosing and treating breast cancer in the context of differing resources.^16^ For example, in countries with highly developed healthcare systems where there is a relatively high incidence of breast cancer, implementation of population-based screening programmes for early detection of asymptomatic disease is a reasonable strategy to reduce mortality. These are, however, expensive and resource intensive and require comprehensive and quality assured cancer services in place to treat detected disease. In lower resource settings, emphasis is appropriately placed on strategies that aim to increase the rates of early diagnosis in symptomatic populations through education, opportunistic screening and improved access to and provision of high quality and affordable cancer care to increase survival from treatable disease.^16^ The fundamental aim of either approach is to downstage cancer diagnosis to improve mortality rates and the cost of treatment.

Several measures are needed to facilitate timely diagnosis and provision of appropriate treatment: this includes tackling some of the financial and logistical barriers of accessing comprehensive cancer care. In low resource settings many healthcare systems require individuals to self-fund large proportions of the health care costs. The costs of investigations and treatment for a cancer diagnosis can result in catastrophic expenditure, potentially pushing households further into poverty. Addressing the financial barriers to healthcare is a key Sustainable Development Goal for health and will be achieved through the widespread implementation of universal health coverage (UHC) which means that “all individuals and communities receive the health services they need without suffering financial hardship”.^17^

A key challenge for developing regions is a lack of publicly funded healthcare facilities for diagnosis and treatment which require a skilled workforce and specialist equipment. The lack of comprehensive radiology and pathology services in low-income countries can result in over- or under-treatment due to inaccurate diagnosis.^18^

Resource stratified guidelines for the diagnosis of breast cancer recommend a minimum of adequate clinical assessment, basic imaging and a tissue diagnosis with fine needle aspiration and/or biopsy. In low resource settings, where populations tend to be younger, ultrasound is an effective low-cost technology to supplement clinical examination and facilitate image guided biopsies. The familiar model of mammogram-based screening which requires expensive equipment that is difficult to transport and maintain, and interpretation by experts, may not be appropriate in less-developed countries.

Digital advances may also alleviate some of these pressures. For example, the shortages of the skilled workforce could be addressed by the creation of centralised reporting hubs and ultimately, artificial intelligence could contribute to reducing this pressure as the clinical safety is established but as yet remains unproven in low resource settings. Accurate diagnosis will support planning of low cost and clinically effective treatment including surgery (modified radical mastectomy) and access to effective drugs on the WHO essential medicine list such as anti-oestrogenic therapy and chemotherapy.

Establishing essential diagnostic and treatment modalities should be a pre-requisite before shifting focus to provide more complex options such as early detection of asymptomatic populations and more sophisticated treatments such as breast conserving surgery and radiotherapy.^19^ In the meantime, lives may be saved by focussing on earlier presentation to downstage breast cancer from advanced disease to a stage where treatments are known to have a beneficial effect on mortality.^20^

## Summary

Breast cancer represents a true global health challenge with considerable unmet medical need. An increasing global burden of breast cancer is unavoidable as incidence rates rise in the less developed regions of the world but poor survival does not need to be inevitable. Sustained and equable improvements in outcomes from this treatable disease require concerted and coordinated initiatives in all parts of the world over the years to come. Future research to identify effective interventions with ongoing evaluation to assess the impact of these efforts is vital to reduce the disparities that are currently observed.
